# Culturable Bacterial Diversity from the Basaltic Subsurface of the Young Volcanic Island of Surtsey, Iceland

**DOI:** 10.3390/microorganisms10061177

**Published:** 2022-06-08

**Authors:** Pauline Bergsten, Pauline Vannier, Julie Frion, Alan Mougeolle, Viggó Þór Marteinsson

**Affiliations:** 1 Matís, Exploration and Utilization of Genetic Resources, 113 Reykjavík, Iceland; paulineb@matis.is (P.B.); pauline@matis.is (P.V.); frion.julie@gmail.com (J.F.); alan.mougeolle@outlook.com (A.M.); 2 Faculty of Life and Environmental Sciences, University of Iceland, 102 Reykjavík, Iceland; 3 Faculty of Food Science and Nutrition, University of Iceland, 102 Reykjavík, Iceland; 4 Agricultural University of Iceland, 112 Reykjavík, Iceland

**Keywords:** oceanic subsurface, culturable microbial diversity, bacteria, extreme environment, Surtsey, Iceland

## Abstract

The oceanic crust is the world’s largest and least explored biosphere on Earth. The basaltic subsurface of Surtsey island in Iceland represents an analog of the warm and newly formed-oceanic crust and offers a great opportunity for discovering novel microorganisms. In this study, we collected borehole fluids, drill cores, and fumarole samples to evaluate the culturable bacterial diversity from the subsurface of the island. Enrichment cultures were performed using different conditions, media and temperatures. A total of 195 bacterial isolates were successfully cultivated, purified, and identified based on MALDI-TOF MS analysis and by 16S rRNA gene sequencing. Six different clades belonging to Firmicutes (40%), Gammaproteobacteria (28.7%), Actinobacteriota (22%), Bacteroidota (4.1%), Alphaproteobacteria (3%), and Deinococcota (2%) were identified. *Bacillus* (13.3%) was the major genus, followed by *Geobacillus* (12.33%), *Enterobacter* (9.23%), *Pseudomonas* (6.15%), and *Halomonas* (5.64%). More than 13% of the cultured strains potentially represent novel species based on partial 16S rRNA gene sequences. Phylogenetic analyses revealed that the isolated strains were closely related to species previously detected in soil, seawater, and hydrothermal active sites. The 16S rRNA gene sequences of the strains were aligned against Amplicon Sequence Variants (ASVs) from the previously published 16S rRNA gene amplicon sequence datasets obtained from the same samples. Compared with the culture-independent community composition, only 5 out of 49 phyla were cultivated. However, those five phyla accounted for more than 80% of the ASVs. Only 121 out of a total of 5642 distinct ASVs were culturable (≥98.65% sequence similarity), representing less than 2.15% of the ASVs detected in the amplicon dataset. Here, we support that the subsurface of Surtsey volcano hosts diverse and active microbial communities and that both culture-dependent and -independent methods are essential to improving our insight into such an extreme and complex volcanic environment.

## 1. Introduction

The oceanic crust represents the largest habitable environment on Earth and one of the least explored ecosystems on our planet [1]. Numerous extremophiles were previously reported in crustal fluids, including thermophilic fermenters, sulfate reducers, and methanogens [2,3,4,5]. Despite the undeniable existence of microbial activity in the oceanic crust [4,5,6,7,8,9,10,11], little is known about the diversity of microbial species from such environments. Advances in molecular techniques, high-throughput sequencing technologies, and bioinformatics have led to major improvements in exploring microbial communities in their environment and provided tools for studying the diversity, distributions, and activities of microorganisms without the need to cultivate them [12]. Such methods resulted in the discovery and characterization of rare biospheres in the most extreme environments, giving precious clues to the metabolic potential and activity of many yet-to-be cultivated microorganisms [12,13,14,15]. Nevertheless, cultivation-dependent methods still remain the best tool for studying a microorganism’s physiology, metabolism, and ecology in ways that cannot be achieved using culture-independent approaches (e.g., [16]). Furthermore, cultivation can offer complementary insights into an ecosystem in combination with molecular tools, although the culturable microorganisms represent a minor component of the actual microbial community [17,18,19,20]. This includes the ecological role of the culturable microorganisms in the environment, their physiological limits, and their adaptive capacities to extreme conditions.

The island Surtsey is a rift zone volcano located in the south of Iceland that emerged from the seafloor between 1963 and 1967 [21,22,23]. In 1979, a cored borehole (SE-01) was drilled through the young volcano for geological exploration [22,24]. The active hydrothermal-seawater system discovered passes through the subsurface deposits, in which the maximal temperature of 124.6 °C in 2017 exceeded the presumed upper limit for functional life [22,25,26,27,28]. Thirty-eight years later, three new cored boreholes (SE-02a, SE-02b, and SE-03) were drilled specifically for microbiological and geological analysis [29,30,31]. Over the years, many studies have explored the Surtsey edifice in terms of geology, mineral, and chemical composition, suggesting that fluid–rock interactions in the submarine Surtsey basaltic deposits behave similarly to basaltic oceanic crust [32,33,34,35,36]. The Surtsey volcano geothermal system can be considered as analog for seawater–oceanic crust interactions that occur at seamounts and in ridge flank systems without the presence of overlying sediments [33]. Surtsey thus serves as a unique natural laboratory for investigating fluid–rock–microbe interactions, and its boreholes can be thought of as opened windows from the surface, allowing for the examination of subsurface microbial processes at high temperatures associated with oceanic crust. Diverse bacterial and archaeal taxa were detected in the subsurface of Surtsey using high-throughput sequencing [27,37]. Although many of these taxa were previously reported in surface and subsurface habitats in both terrestrial and marine settings, many of the newly discovered clades belonged to previously unknown lineages [37].

In this study, we used both specific and non-specific culture media under various temperature conditions to enrich high diversity of microorganisms from the subsurface of Surtsey, which includes fumarole, borehole fluid, and drill core samples. The objectives were (i) to describe the subsurface culturable microbial diversity; (ii) to determine if the application of a diverse range of media, temperature, and cultivation methods can enable the isolation of subsurface microorganisms previously only detected through culture-independent analysis; and (iii) to contribute to the understanding of the cultivable diversity of extreme subsurface environments, especially in the warm and newly formed oceanic crust.

## 2. Materials and Methods

### 2.1. Study Site and Sample Collection

The sampling site, Surtsey island (63°18′10.8″ N; 20°36′16.9″ W), is located approximately 35 km from the south coast of Iceland, within the southern offshore extension of Iceland’s Eastern Volcanic Zone. All samples were collected on the island between 2016 and 2018. The hydrothermal system was active with a maximal temperature of 124.6 °C at 100 m depth in 2017 [24,26,27,28]. Three sample types were collected for cultivation purposes (Table 1): (i) drill core samples collected at successive depths from SE-02a and SE-02b, (ii) borehole fluids from the four drill holes (SE-01, SE-02a, SE-02b, and SE-03), and (iii) condensed steam and biomass from fumarole outlets located on the two tephra cones (63°18′15.4″ N 20°36′07.7″ W and 63°18′19.9″ N 20°36′24.7″ W). All samples were immediately processed aseptically on-site and kept at 4 °C [30,37].

Drill cores were sampled during the ICDP SUSTAIN drilling operation at Surtsey in 2017 [30,37]. At the drill site, an 8 cm section was cut from every third 3 m core run at 70 cm from the top and was immediately removed from the liner, put into a sterile plastic bag, oxygen-removed by GasPak™ (BD), and stored at 4 °C. In the laboratory, drill core samples were fragmented with a hammer into an anaerobic chamber (atmosphere: N_2_/CO_2_/H_2_: 80/10/10, Coy Laboratory Inc., Grass Lake, MI, USA), and interior fractions were split into smaller pieces in a mortar. All tools for crushing were autoclaved, disinfected by ethanol, and flamed before and between each use. Two cultivation methods were tested with the drill core samples. Some basaltic samples were transferred into artificial seawater (ASW) supplemented by Wolin’s vitamin solution (DSMZ, medium 141) (1X) [38]. After being shaken overnight and stored at 4 °C for sedimentation, the supernatant was used for cultivation. In addition, small pieces of basalt were directly transferred into media for enrichment. Borehole fluid samples were collected using a stainless-steel bailer, as previously described in [27]. Steam from fumaroles was collected over 12 h of continuous sampling by introducing a sterile rubber hose into the outlet of the fumarole with the other end connected to a sterile plastic container. Both borehole fluids and steam, with were condensed into water, from fumarole were aliquoted, reduced by Na_2_S solution (0.05% *w*/*v* final concentration), and stored at 4 °C. In addition, biomass samples (mud and dead flies) were collected aseptically near the outlet fluxes and were stored at 4 °C in falcon tubes in anaerobic conditions using GasPak™ (BD).

### 2.2. Media Preparation, Enrichment, and Strains Isolation

Seven different culture media were tested, non-selective and selective (e.g., media for methanogens, iron, sulfate, and sulfur reducers), at various temperatures (i.e., 22 °C, 40 °C, 60 °C, and 80 °C) and under both aerobic and anaerobic conditions.

Aerobic cultures were performed on plates and in liquid non-selective media for marine heterotrophs (MB: Marine Broth 514 medium, BD Difco™) at pH 7 and 9 and in medium 166, a standard medium for aerobic thermophiles [39], supplemented with 2% NaCl (Table S1). Two jellifying agents were used: agar (14 g/L, Sigma-Aldrich, Deissenhofen, Germany) for incubation temperature below 60 °C or Phytagel™ (8 g/L, Sigma-Aldrich, Deissenhofen, Germany) for incubation temperature above 60 °C. 

Anaerobic cultures were prepared in liquid selective media containing modified ASW, with the addition of different substrates (solution A) (Tables S2–S5). Those media were tested with different salinities (1 to 3% (*w*/*v*) NaCl). The modified ASW basis contained per liter of distilled water: NaCl (10 to 30 g), NH_4_Cl (0.5 g), MgSO_4_·7H_2_O (3.4 g), MgCl_2_·6H_2_O (4.18 g), KCl (0.33 g), FeSO_4_·7H_2_O (0.01 g), Na_2_SeO_3_·5H_2_O (1 mg), and PIPES buffer (3 g). Before being autoclaved, a few drops of resazurin (0.1% *w*/*v*) were added to the modified ASW as an indicator of O_2_ variations and substrates were differentially added to the different media as follows (per liter): the medium for sulfur-reducing microorganisms (modified YPS, Table S2) contained a yeast extract (0.5 g) and peptone (0.5 g), and pH was adjusted to 7; the medium for sulfate-reducing microorganisms (SO, Table S3) was supplemented by Na_2_SO_4_ (0.2 g), a yeast extract (0.2 g), L-lactate (0.5 g), Na-pyruvate (0.5 g), and L-ascorbate (0.5 g), and pH was adjusted to 7.5; the medium for iron-reducing microorganisms (I, Table S4) contained Fe (III) citrate (10 g) and Na-acetate (0.1 g), and pH was adjusted to 8; and the medium for methanogens (M, Table S5) was supplemented by a yeast extract (1 g), and pH was adjusted to 7.5.

In parallel, solutions B (K_2_HPO_4_ at 2.8% *w*/*v*) and C (CaCl_2_·2H_2_O at 10% *w*/*v*) were prepared and autoclaved separately. A trace elements solution (DMSZ 141 medium) was prepared as well and was filtrated-sterilized (0.22 µm filter). After sterilization and cooling, the solutions were added to solution A as follows: 5 mL of solution B, 5 mL of solution C, and 10 mL of the trace element solution. In addition, 10 g of elemental sulfur and 10 mL of the filtrated-sterilized solution of vitamins (DMSZ 141 medium) were added into the medium YPS (Table S2); 20 mL of NaHCO_3_ (0.1% *w*/*v*) was added into the medium SO (Table S3); 10 mL of a filtrated-sterilized solution of vitamins (DMSZ 141 medium), 20 mL of NaHCO_3_ (0.2% *w*/*v*), and 0.25 mL of Na_2_WO_4_·2H_2_O (0.1% *w*/*v*, N_2_) were added into the medium for I (Table S4); and 10 mL of a vitamin solution (DMSZ 141 medium), 20 mL of NaHCO_3_ (0.2% *w*/*v*), 0.5 g/L of coenzyme M (2-mercaptoethanesulfonic acid), and 5 mL of methanol were added into the medium M (Table S5).

All media were supplemented with a gas phase of H_2_/CO_2_ (80/20, 1.5 to 2 bars), except medium SO, which was supplemented with a gas phase of N_2_ (100, 1.5 to 2 bars). All media were reduced with a sterile Na_2_S·9H_2_O solution (0.05% *w*/*v*, pH 7, N_2_) with the addition of a L-Cysteine-HCl·H_2_O solution (0.05% *w*/*v*, pH 7, N_2_) in the medium M.

In the dark, culture media were inoculated with 1 to 3% of the basaltic suspension, small bits of rock, fumarole biomass fragments, or 1% of fluid samples (borehole fluid and condensed water from fumarole). The uninoculated culture media were incubated for each medium and incubation temperature under the same conditions as the negative controls. After growth was observed in the enrichment cultures, the plates were directly inoculated with the enrichment culture as an inoculum. If no growth was observed on the plate after 5 days, the dilution-to-extinction technique was employed. Growth was monitored by colony observation on plates or under the microscope (Olympus BX51 at 100X/1.30 Oil pH3). Colonies with unique morphological features were selected and were streaked at least six times before being considered pure. Using the dilution-to-extinction technique for strain isolation in liquid cultures, pure cultures were obtained from the highest positive dilution tube [40].

In total, 195 isolates were added to the Icelandic Strain Collection and Records (ISCaR) and stored at −140 °C (Table 2). Glycerol (20% *v*/*v*) was used as cryoprotectant for aerobic isolates, and two different preservation methods were used for anaerobic isolates—glycerol (20% *v*/*v*) and dimethyl sulfoxide (DMSO) (0.025% *v*/*v*).

### 2.3. Identification of Isolates by 16S rRNA Gene Sequencing

Matrix-assisted laser desorption/ionization time-of-flight mass spectrometry, MALDI-TOF MS (Microflex LT, Bruker Daltonics, Bremen, Germany), was used for strain differentiation, allowing for the selection of colonies to be sequenced. Each colony was extracted using a standard formic acid/acetonitrile procedure. In brief, a full loop of fresh culture was diluted in 300 µL of autoclaved milliQ water and 900 µL of ethanol. After centrifugation and elution of ethanol, the pellet was dried. Then, depending on the size of the pellet, a volume between 5 and 30 µL of a 70% formic acid solution was added, followed by the same volume of acetonitrile. After centrifugation, 1 µL of the supernatant was spotted on the target plate using a saturated α-cyano-4-hydroxycinnamic acid (HCCA) matrix solution. Measurements were consistently carried out using the same instrument parameters.

In parallel, single colony DNA was extracted from pure cultures and was carried out using a fresh 6% Chelex^®^ 100 solution, as previously described [41]. DNA was quantified with a NanoDrop^®^ ND-1000 UV-Vis Spectrophotometer (NanoDrop Technologies, Inc., Wilmington, DE, USA). From the selected isolates, partial sequences of the 16S rRNA gene were amplified using forward primer F9 (“5-GAGTTTGATCCTGGCTCAG-3”) and reverse primer R805 (“5-GACTACCCGGGTATCTAATCC-3”) [42]. PCR was performed using OneTaq^®^ Hot Start DNA Polymerase (New England BioLabs Inc. (NEB), Ipswich, MA, USA; #M0481L), according to the recommendation of the manufacturers. The PCR program started with an initial denaturation step at 94 °C for 30 s, followed by 35 cycles of denaturation at 94 °C for 30 s, annealing at 52 °C for 1 min, and extension at 68 °C for 1 min. A final extension at 68 °C for 5 min was also included. The negative control was always used to exclude contamination. The amplification products were confirmed by agarose gel electrophoresis (1%) stained with SYBR^®^ Safe DNA Gel Stain (Thermo Fischer Scientific, Waltham, MA, USA; #S33102) for 40 min at 100V and 400 mA in a 1X TAE buffer (2 M Tris, 1 M acetic acid, 50 mM EDTA disodium salt). PCR products were purified using Exonuclease I (ExoI, NEB, Ipswich, MA, USA; #M0293S), Shrimp Alkaline Phosphatase (SAP, NEB, Ipswich, MA, USA; #M0289S) and were sequenced with an ABI 377 DNA sequencer using a BigDye Terminator Cycle Sequencing Ready Reaction kit according to the manufacturer (Perkin-Elmer Applied Biosystems, Foster City, CA, USA). The quality and analysis of partial 16S rRNA gene sequences obtained from the isolates were checked with the software Sequencher 5.2.4 software (Gene Codes Corp., Ann Arbor, MI, USA), and closely related sequences were identified using BLASTn (Basic Local Alignment Search Tool) at NCBI (National Center for Biotechnology Information) and the 16S ribosomal RNA (Bacteria and Archaea type strains) database. 

### 2.4. Construction of Phylogenetic Trees

One or two representative 16S rRNA gene sequences of each taxonomic group (55 sequences) were selected to build the phylogenetic tree. Sequences were classified and aligned using the online portal of the SILVA Incremental Aligner (SINA 1. 2. 11) tool of the ARB-Silva database (http://www.arb-silva.de/aligner/, accessed on 7 July 2021) [43]. The SILVA reference alignment searched the related sequences (two nearest neighbors per sequence) to 90% min identity of the 16S rRNA gene sequences from this study. Columns containing 10% or less rows of sequences were stripped of the full alignment, generating a final alignment with 158 taxa (55 from this study) and 1571 nucleotides. Maximum likelihood analyses were carried out using RAxML BlackBox (https://raxml-ng.vital-it.ch/#/, accessed on 13 September 2021) [44], as implemented on the CIPRES webserver [45] under the GTR GAMMA model. The tree in NEWICK format was imported into Interactive Tree Of Life (iTOL) v6.4 [46]. An additional tree that aligned all the sequences (151) with 442 sequences from the ARB-Silva database can be found in the Supplementary Materials (Figure S1).

Seven representative sequences of the novel strains (<98.65% 16S rRNA gene sequence similarity) isolated in this study were selected and aligned against 16S rRNA gene sequences of the closest cultured type strains using the clustal_w program [47]. Phylogenetic trees were reconstructed using the neighbour-joining tree algorithm [48] in MEGA7 [49], based on 1000 bootstrap replications and a total of 224 positions in the final dataset.

### 2.5. Bacterial Cultured Collection vs. 16S rRNA Amplicon Gene Sequencing

The 16S rRNA amplicon datasets used for the comparison with the sequences of the isolated strains were previously published in the European Nucleotide Archive (ENA) at EMBL-EBI (accession number ERP126178) [37]. Briefly, the datasets were obtained by Illumina MiSeq paired-end (2 × 300 base pair) tag sequencing using the universal primers 515f (5′-GTG CCA GCM GCC GCG GTA A-3′) and 806r (5′-GGA CTA CHV GGG TWT CTA AT-3′) [50]. Sequence variants were inferred using the R Package DADA2 [51] version 1.4, as described elsewhere [37], and the SILVA SSU database release 138 was used for taxonomic assignation [43]. A phyloseq object [52] was constructed directly from the DADA2 outputs and the Amplicon Sequence Variant (ASV) table before any contaminant removal was used for the alignment. The comparisons between the ASVs from 16S rRNA amplicon gene sequencing datasets and partial 16S rRNA gene sequences obtained by Sanger sequencing were performed using BLASTn optimized for highly similar sequences (megablast). All ASVs were basted against the 55 representative sequences of the isolated strains (Figure S2). 

## 3. Results

### 3.1. Cultivated Bacterial Diversity

**Figure 1:**
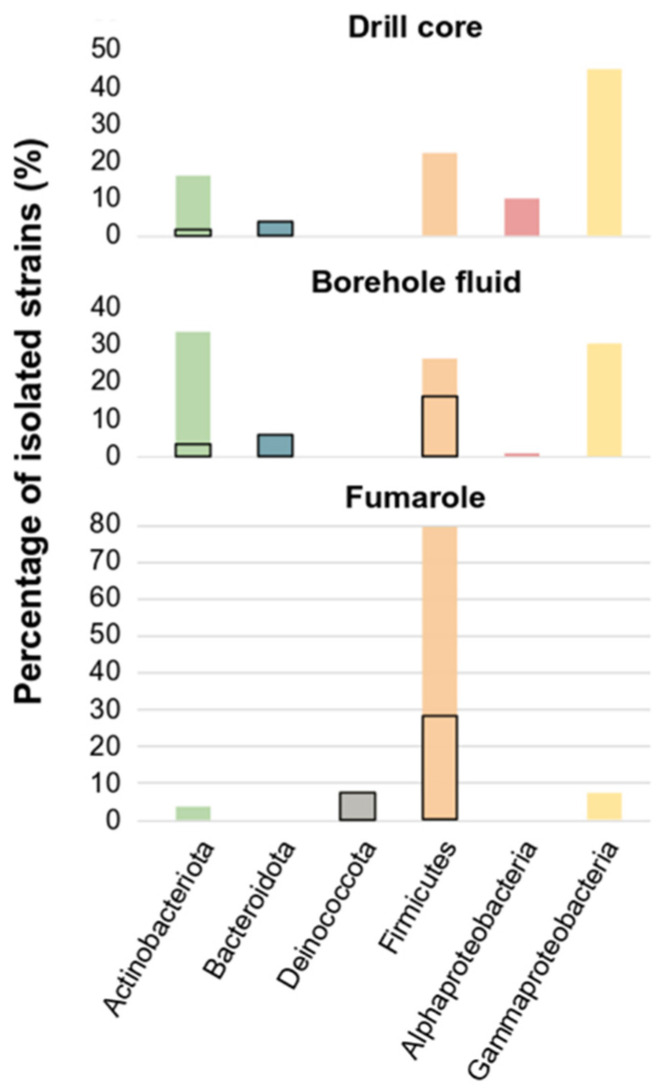
Taxonomical distribution at the phylum level (classes for Proteobacteria) of the bacterial isolates from Surtsey island by sampling sites. The percentages represent the relative cultivable bacterial abundance. Thermophilic strains (≤60 °C) are framed in bold.

Under the microscope, 151 of the 270 enrichment cultures in the tested media and temperatures indicated growth. Positive growing enrichments were used as an inoculum in a subsequent medium. The isolates were obtained after either randomly selecting colonies that appeared on plates or after using the dilution-to-extinction method [40]. We obtained 195 isolates of bacteria from fumarole samples, borehole fluids, and drill cores collected from Surtsey island (Table 1). Most of the isolates were grown on the culture media 166 (101 strains), at 22 °C (120 strains), and in aerobic conditions (148 strains). The highest number of isolated strains came from borehole fluid samples (49.23%), followed by fumarole (26.15%) and drill cores (24.62%) (Figure 1 and Table 2).

Although many enrichment cultures showed growth in anaerobic conditions, subsequent growth was unsuccessful and most of the isolates were aerobic and heterotrophic bacteria. Thermophilic strains represented almost 30% of the strain collection, with 58 strains isolated at temperatures above 60 °C.

The isolates were identified by comparative analysis of their partial 16S rRNA gene sequences against the NCBI reference sequences (RefSeq) database. They were assigned to different families of five bacterial phyla (Figure 1), representing 42 different genera (Table 2 and Table S6). No Archaea isolates were obtained in pure culture under the culture conditions tested. The isolates were assigned to Actinobacteriota (43 strains), Bacteroidota (8 strains), Alphaproteobacteria (6 strains), Gammaproteobacteria (56 strains), Deinococcota (4 strains), and Firmicutes (78 strains) (Table 2). Firmicutes were mostly isolated from fumarole samples (80%), Gammaproteobacteria were mostly isolated from the drill core samples (46%), and Actinobacteriota were mostly isolated from borehole fluid (35%) (Figure 1). Most of the thermophilic strains belonged to the phylum Firmicutes, while eight strains of thermophilic Bacteroidetes and four strains of thermophilic Actinobacteria were isolated. In addition, four strains were isolated at 80 °C, and all belonged to the phylum Deinococcota. Only strains that belong to Rhodobacteraceae, Enterobacter, Micrococcus, and Microbacterium were isolated on the medium SO, while one strain of Sphingomonadaceae was isolated on the medium YPS. Enrichment cultures targeting iron, sulfur, sulfate reducer microorganisms, and methanogens showed growth, but only *Cryobacterium*, *Frigoribacterium*, *Leifsonia*, *Acinetobacter*, *Micrococcus*, and species that belong to the Enterobacterales were isolated on the media M and I and the rest of the enrichments could not be maintained by the dilution-to-extinction technique to isolate strains.

### 3.2. Phylogeny of the Isolates and Habitat of the Closest Relatives

**Figure 2:**
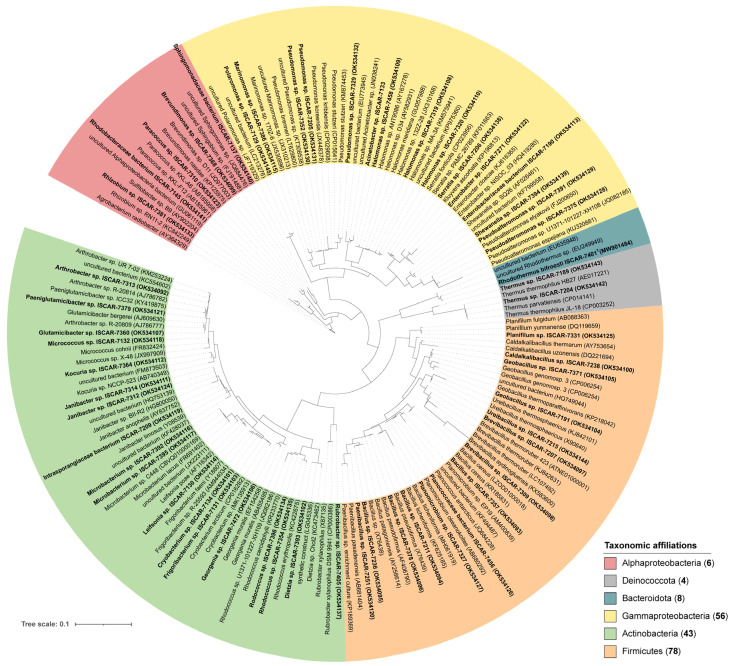
Maximum likelihood phylogeny of the partial 16S rRNA gene sequences placing the cultured bacteria from the subsurface of Surtsey island. The final alignment contained 158 sequences, 55 from this study (in bold), and was generated using the SILVA SINA alignment tool and the SILVA reference alignment. The tree was constructed using RAxML under the GTR GAMMA model of evolution.

The 16S rRNA genes of the isolates were partially sequenced. After trimming the end of the sequences to increase quality, the sizes of the sequence were mostly between 400 and 600 bp and sequence quality along all sequences was above 90%. Few sequences were smaller than 400 bp and thus were unambiguously assigned at the family level. To overcome this, taxonomic assignment of partial 16S rRNA gene sequences obtained from the 55 representative sequences of the isolates was performed using both the BLASTn on NCBI using the RefSeq database and SILVA Incremental Aligner (SINA 1. 2. 11) tool with Silva database version 138.1 [43] (Figure 2 and Figure S1, and Table S6).

**Figure 3:**
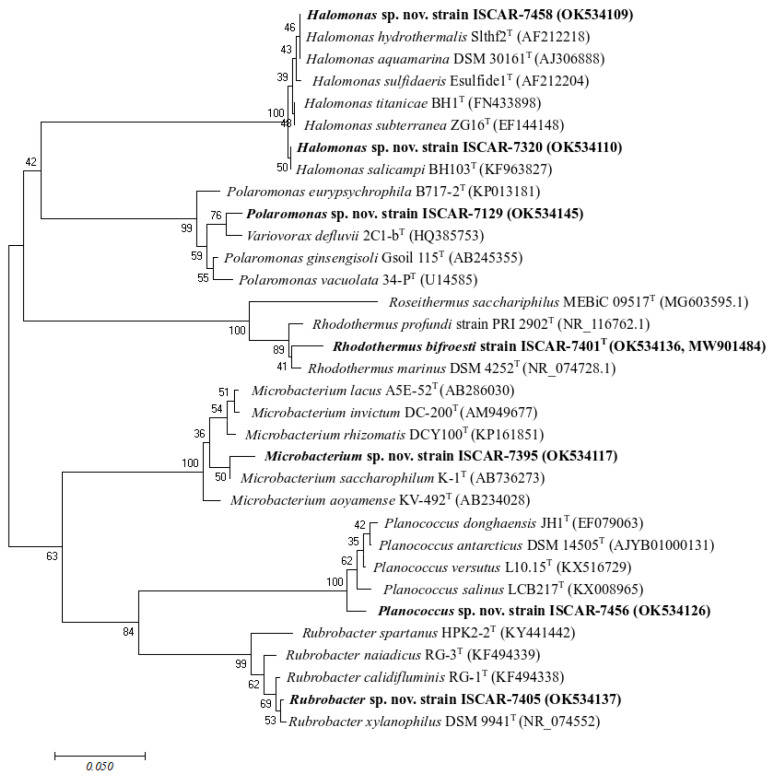
Phylogenetic tree based on partial 16S rRNA gene sequences showing the relationship between the novel species isolated in this study and closest cultured type strains. GenBank accession numbers are given in parentheses. The tree is based on the clustal_w and the neighbour-joining method with 1000 bootstraps using a total of 224 positions in the final dataset. Bar, 0.05 represented the nucleotide substitution per position.

Few isolated strains obtained in this study potentially represent novel species, according to the percentage value used as a boundary for species delineation of 98.65% 16S rRNA sequence similarity [53,54,55]. Twelve thermophilic strains belonging to the Actinobacteriota and the Bacteroidota showed sequence similarity below 98% and were related to *Rhodothermus marinus* (NR_074728) (95 to 96.41% sequence similarity) and *Rubrobacter xylanophilus* (NR_074552) (96.75–97.57%). The closest neighbor sequences corresponded to uncultured *Rhodothermus* species detected in seawater (EU249949) and *Rubrobacter xylanophilus* DSM 9941 isolated from hot spring water samples (CP000386) (Figure 2). The novel strains of *Rhodothermus* were characterized, and the name *Rhodothermus bifroesti* was attributed to the new species [56]. In addition, other isolated strains potentially represent novel species. Their sequences fell close to 98% of sequence similarity and were related to the genera *Planococcus* (97.80%), *Halomonas* (97.87–98.18%), *Microbacterium* (98.11–98.97%), and *Polaromonas* (98.01%) (Figure 3, Table S6). The closest neighbor sequences were detected in permafrost soil (JQ684228), seawater and deep-sea sediment (KP975360, AY582931), soil or continental Antarctic lake (CBVQ010000169, FR691402), and glacial sediment (JF719329) (Figure 2).

Other thermophilic strains were isolated in this study and showed sequence similarity percentages higher than 98%, with the closest related sequences usually detected from thermal environments. They belonged to the genera *Thermus* within the phylum Deinococcota and to *Geobacillus*, *Ureibacillus*, *Brevibacillus*, *Caldalkalibacillus*, and *Planifilum* within the Firmicutes. The only strains isolated at 80 °C were closely related with *Thermus thermophilus* (NR_113293.1), a hyperthermophilic bacterium that was originally isolated in saline hot spring [57]. The closest neighbor sequence was detected in a hydrothermal vent (AE017221) (Figure 2). Three strains showed 99.62% sequence similarity with *Geobacillus subterraneus* (NR_025109.1, NR_132400.1), a hydrocarbon-oxidizing thermophilic bacterium isolated from a petroleum reservoir located at 1200–2730 m b.s. [58]. Other strains isolated in this study were identified as *Geobacillus* sp. and fell into the *Geobacillus* thermoleovorans group. They were closely related to *Geobacillus thermoparaffinivorans* (KP218042), previously isolated from a crude oil deep reservoir [59]. Seven non-thermophilic isolated strains identified as *Paeniglutamicibacter* sp. were also closely related to sequences previously detected in oil-brine from an oilfield reservoir (NR_026237.1). Four strains were closely related to *Ureibacillus thermosphaericus* isolated from the air (NR_119203.1, X90640), while six strains were identified as *Brevibacillus* spp. and showed a high sequence similarity percentage with species previously isolated from hot spring (NR_117986.1) or hot water (ATNE01000001). One isolated strain was closely related at 99.93% sequence similarity to *Planifilum yunnanense* (NR_043563.1), a thermophilic thermoactinomycete that has been isolated from hot spring [60]. In addition, six other thermophilic strains isolated from borehole fluids showed 99.45% sequence similarity with *Caldalkalibacillus uzonensis* (NR_043653.1; DQ221694), an alkali-thermophilic bacterium isolated from hot spring [61]. 

Non-thermophilic strains isolated in this study were also closely related to alkaliphilic bacteria. Their sequences were closely related to *Dietzia natronolimnaea* (NR_116683.1), which has been isolated from soda lake [62], and to *Bacillus pseudofirmus* (X76439, AF406790, NR_026139.1) and *Bacillus patagoniensis* (AY258614, X76438, NR_025741.1), which were both recently reclassified to the genus *Alkalihalobacillus* [63]. 

Twenty-six isolates were closely related to species previously reported in marine habitats, including deep-sea sediments. These comprised *Planomicrobium* (NR_113593.1, AB680292), *Paracoccus* (NR_113921.1, AB185961), *Pseudoalteromonas* (NR_044837, FJ200650), *Sulfitobacter* (NR_043547.1, AY902204), *Marinomonas* (NR_116234.1, JX530896, JX310213), *Shewanella* (NR_040951.1, KF799558), and *Halomonas* (NR_116997.1, NR_027185.1, KP975360, AY582931) (Figure 2). 

Few isolated strains identified in this study belonging to the Intrasporangiaceae family were closely related to *Janibacter limnosus* (NR_026362.1). Although this species has been previously reported as endemic from deep-sea sediments along with *Rhodococcus*, *Arthrobacter*, *Kocuria*, and *Dietzia* [64], the closest neighbor sequences were detected in a wastewater treatment plant (Y08539) and metazoan gut’s (HQ753139, AY837752). While some of the isolated strains were closely related to sequences that could be potential contaminants (e.g., *Paenibacillus pasadenensis* (AB681404), *Rhodococcus* from group 2 (KC422675), *Glutamicibacter bergerei* (AJ609630), and *Micrococcus cohnii* (FR832424, NR_117194.1)), other strains isolated in this study were closely related to sequences that were previously detected in the environment. This involved the permafrost (e.g., *Rhodococcus cercidiphylli* (KR233770), and *Cryobacterium arcticum* (CP016282, MK135913) and soil (e.g., uncultured *Arthrobacter* (KM253224, KC554602, NR_041546.1), uncultivated *Kocuria* (FM873503), *Leifsonia poae* (AF116342), *Bacillus licheniformis* (MH061919), *Frigoribacterium faeni* (Y18807), *Rhizobium* sp. RN11-2 (KC842249), and *Brevundimonas* sp. D11 (JQ977003)). 

Overall, our results showed that many of the isolated species were closely related to sequences previously reported in marine (e.g., seawater, sediment, and deep-sea hydrothermal vent), alkaline (e.g., submarine alkaline or terrestrial hot springs, soda lake, and salty soil), and subsurface environments (e.g., petroleum reservoirs), while others showed percentages of 16S rRNA gene sequence similarity below 98%, suggesting that they might represent novel species.

### 3.3. Comparison of the Bacterial Diversity Observed by Amplicon Sequencing from Environmental Samples and Cultured Diversity

**Figure 4:**
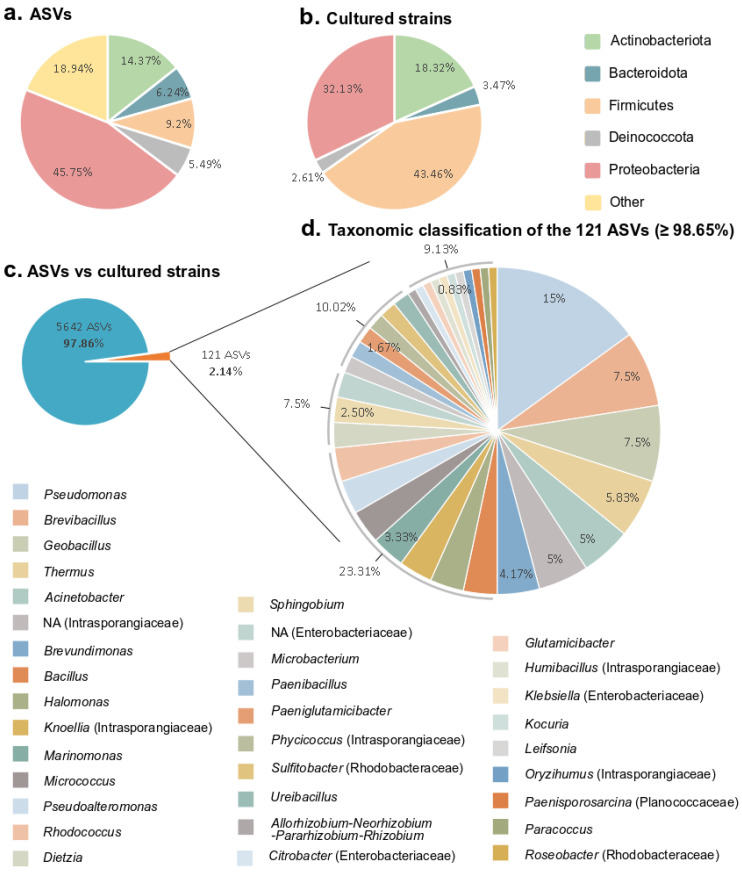
For each phylum, number of ASVs obtained by high throughput sequencing (**a**) and cultured strains (**b**). (**c**) The fraction of the in situ diversity represented by cultured strains, and taxonomic classification (**d**). An ASV was considered to be represented by the cultured strains if the ASV had equal or higher than 98.65% sequence similarity with the 16S rRNA gene sequence of cultured strains. The taxonomic classification of the ASVs represented by the cultured strains at the genus level. Family given in parenthesis for Enterobacteriaceae, Intrasporangiaceae, Planococcaceae and Rhodobacteraceae. NA. Not assigned genera.

The culture-dependent (isolation) and -independent (metabarcoding) approaches showed different taxonomic distributions at the phylum level (Figure 4a,b). As expected, the number of phyla obtained by the culture-dependent approach was much lower than observed using the molecular method. Of the 49 phyla detected in the 16S rRNA gene amplicon datasets, only 5 were recovered in the culture collection (Figure 4a,b). However, those five phyla represented more than 80% of the ASVs obtained by amplicon sequencing (Figure 4a). 

To determine if the cultured strains were detected in the subsurface of Surtsey island, we used a culture-independent method (Figure S2). The partial 16S rRNA gene sequences of the isolates were aligned against Amplicon Sequence Variants (ASVs) from the 16S rRNA gene amplicon datasets (accession number ERP126178), which were obtained from the same samples as for cultivation [37] (Figure S2). Only the ASVs matching at percentages higher than 98.65% sequence similarity with sequences from the cultured strains were kept for investigation (Figure 4d and Table 3; bold values). This threshold was selected for species delimitation, assuming that the same species was detected using culture-dependent and -independent methods at an equal or higher percentage. Among a total of 5642 unique ASVs from the amplicon datasets, only 121 ASVs (≥98.65% sequence similarity) were represented by the culturable strains, which correspond to 2.14% of the in situ diversity (Figure 4c). This fraction belongs to 34 different genera, such as *Pseudomonas*, *Brevibacillus*, *Geobacillus*, *Thermus*, *Acinetobacter*, a non-assigned genus from the Intrasporangiaceae family, and *Brevundimonas*, accounting for half of the 121 ASVs (Figure 4c,d). This suggests that some of the species isolated in this study were also detected in the subsurface of Surtsey island using high-throughput sequencing.

Table 3 indicates in which sample type the 121 ASVs were detected, in addition to the samples origin of the isolates. The sample types correspond to the 16S rRNA amplicon gene sequencing of fumarole, borehole fluid, and drill core samples (Table 3, F, BF, and DC) as well as the sequencing of blank as extraction controls (Table 3, Control). For example, ASVs detected in the borehole fluid and drill core samples possibly represent the same species as the *Dietza* sp. isolated in this study from a drill core sample (Table 3). Likewise, other cultured species were detected in the samples using a culture-independent approach. These species include *Kocuria*, *Leifsonia*, *Paeniglutamicibacter*, and *Rhodococcus* from group 2; *Bacillus* (*para*)*licheniformis*, *Brevibacillus*, *Geobacillus*, and *Geobacillus* from the thermoleovorans group; and *Paenibacillus*, *Ureibacillus*, *Allorhizobium*-*Neorhizobium*-*Pararhizobium*-*Rhizobium*, *Paracoccus*, *Sphingobium*, *Halomonas*, *Marinomonas*, and *Pseudomonas* from groups 1 and 3; and the cultured strains that belong to the family Rhodobacteraceae (Table 3). The latter species were not detected in the blank extraction controls (Table 3; Control), indicating that they might be endemic to the subsurface of the island.

Surprisingly, we cultivated genera that were not detected using metabarcoding (≤98.65%). They belong to *Arthrobacter*, *Georgenia*, *Rubrobacter*, *Rhodothermus*, *Caldalkalibacillus*, *Planifilum*, *Planomicrobium*, *Polaromonas*, *Serratia*, and *Shewanella*. Indeed, if no ASVs matched with a cultured strain sequence above 98.65%, ASVs with the highest score were kept in Table 3 as an indication of the closest taxa (Table 3, light values). Two species isolated in this study, *Georgenia* and *Planifilum*, did not match any detected ASVs, while other isolated species aligned with ASVs at low sequence similarity. These include *Arthrobacter* (97.78%), *Rubrobacter* (93.6%), *Rhodothermus* (95.58%), *Bacillus* from group 1 (97%), *Caldalkalibacillus* (98.53%), *Polaromonas* (98.27%), *Shewanella* (96.7%), and the cultured strain from the Planococcaceae family (97.67%).

Many of the species isolated in this study matched at high sequence similarity percentages with ASVs detected in the extraction blanks. These include *Microbacterium lacus*, *Micrococcus*, and *Rhodococcus* from group 1; *Thermus*, *Caldalkalibacillus*, *Brevundimonas*, *Acinetobacter*, *Polaromonas*, *Pseudoalteromonas*, and *Pseudomonas* from group 2; and the cultured strains belonging to the families Intrasporangiaceae and Enterobacteriaceae. While those taxa were detected in the extraction blanks, they were frequently detected at a much higher abundance in the samples. In contrast, the strain identified as *Glutamicibacter* sp. was only detected in the extraction blanks at 100% sequence similarity, suggesting that it is a potential contaminant.

## 4. Discussion

Most of the isolates cultured in this study were highly related to aerobic bacteria, and only a few taxa were isolated under anaerobic conditions. Among the 270 enrichment conditions tested, two nutrient-rich media, 166 and MB, were the most successful in the isolation of strains (Table 2). No archaea were isolated, and all of the bacterial isolates were heterotrophic. Our results suggest that the media and conditions tested were ineffective for isolating archaea and chemolithoautotrophs, although those taxa were detected in the subsurface of the Surtsey volcano [37]. The vast majority of the bacterial diversity in our samples seems to be uncultivable with our cultivation techniques, as it has been previously reported [17,19,20,65]. Archaeal cultivation is even more challenging [66]. Our results are comparable with other findings and could be explained by insufficient care during sampling, transportation, incorrect storage of the samples used as inoculum, or inadequate cultivation conditions and isolation methods [67]. In this study, many isolates could not be maintained or subsequently cultivated, especially colonies obtained in anaerobic conditions. Colonies obtained using the Hungate roll-tube technique [68,69] that could not grow after being transferred on solid or liquid media represent a good example. This could be due to a lack of information on the habitat’s physicochemical parameters, which are necessary for establishing cultivation settings that mimic in situ environmental conditions. For example, trace micronutrient substances or growth factors originally present in the samples could be essential for growth. The utilization of 0.22 µm filtered borehole fluids for media preparation may have thus facilitated the second transfer of colonies and aided in the maintenance of the subcultures. However, the volume of sterilized fluids was insufficient to serve as cultivation medium basis. In addition, some microorganisms can only be cultivated in the presence of other cells. Syntrophic relationships, competition, or inhibition between microorganisms can also exclude the cultivation and isolation of specific taxa. Lowering the nutrient content of the rich media used for enrichment could have improved the diversity of culturable microorganisms (e.g., [70]). In this study, fast-growing bacteria may have been favored due to the nutrient-rich substrate and short incubation time. The multiplication of experiments, methods (e.g., acclimation step, use of antibiotics, innovative cultivation techniques for co-cultivation, simulation of the natural environment, or single-cell isolation), media (wide range of carbon and energy sources), and culture conditions (e.g., temperature and pressure) may overcome these challenges.

Closest relatives to our isolated strains were previously detected in soil, seawater, and geothermal active sites near the surface (e.g., hot springs and hydrothermal vents) (Figure 2). This observation supports the hypothesis based on 16S rRNA gene amplicon sequencing results that microbial cells from the surrounding environment are transported into the subsurface of Surtsey island by fluid inflow (meteoric water and seawater inflow) and that the colonization of young basalt has been driven by the ability of some microorganisms to adapt to a new environment and to disperse across ecosystems [37]. Our culture-based approach brings insight into the question of whether the cells die, survive, or adapt to the extreme environmental conditions that exist within the volcano since seawater bacteria were alive and cultured from borehole fluid or drill core samples (e.g., *Pseudoalteromonas*, *Halomonas*, and *Marinomonas*, Table 3). In addition, alkaliphilic bacteria (e.g., *Thioalkalimicrobium*) were previously detected in the subaerial deposits of the Surtsey volcano [37]. The isolation of alkaliphilic bacteria (e.g., *Caldalkalibacillus*) from the subsurface of Surtsey confirmed that the environmental conditions occurring at some depths are highly saline and alkaline. This could be the result of the biotic or abiotic dissolution of some minerals or basaltic glass at low fluid–rock ratios [33]. It suggests that these groups of adapted microorganisms are present and active within the high-temperature geothermal system of Surtsey (range of 50–150 °C) and could have significant impacts on subsurface geochemistry [71].

As expected from other environmental microbiome research, a disparity was observed between the microbial communities identified in the samples using cultivation-independent methods in comparison with the cultured strains [72]. Among the total ASVs detected using a culture-independent approach (metabarcoding), only 2.14% were recovered in the cultured collection (Figure 4). The threshold of 98.65% sequence similarity was selected for species delimitation. Because some species share a high level of 16S rRNA gene sequence similarity (>99%), it is challenging to differentiate two species using 16S rRNA gene sequences alone. However, many comparative studies investigated large datasets to determine an optimal 16S rRNA gene sequence similarity threshold for microbial species demarcation, considering DNA–DNA hybridization (DDH) and average nucleotide identity (ANI) values, and taking into account the effect of the taxonomic group [55]. These studies suggested that 98.65% of 16S rRNA gene sequence similarity can be used as an adequate threshold for differentiating two species. The high proportion of ASVs detected in subsurface environment samples that remained uncultured could be explained by several reasons. The DNA detected in the samples might come from dead cells, prohibiting their detection using culture-dependent methods [67]. Additionally, as previously mentioned, the cultivation methods applied may be unsuitable for the cultivation of specific microbial taxa, and the application of unappropriated elements such as the composition of the medium, temperature, and oxidative stress can proscribe their growth [73]. Therefore, culturing efforts to characterize microbial communities should include a variety of methodologies and media. Still far too few microorganisms were isolated from extreme environments, especially autotrophic anaerobic microorganisms discovered using molecular techniques. The number of previously uncultured taxa may be increased with the application of innovative culturing methods, the addition of growth factors in the media, and the use of in situ cultivation methods in their original habitats [74,75,76]. At the same time, some of the cultivated genera were not detected with a culture-independent approach and could represent members of the rare biosphere. For example, *Planifilum*, which belongs to the Thermoactinomycetaceae family, was isolated from borehole fluid but was not detected using metabarcoding (Table 3). This observation has been made in a variety of environments [77,78] and suggests that cultivation not only provides a small percentage of what was detected in the sequencing data but also could increase the total diversity present in a sample.

When investigating low biomass samples from subsurface environments using a molecular approach, the incorporation of contaminants is very high during sample collection and processing [79,80]. Based on the datasets obtained through the activity of the Census of Deep Life (CoDL) and the Deep Carbon Observatory on low biomass subsurface environments, a list of genera was published and identified as “typical” potential contaminants that were usually associated with molecular reagents or potential human contamination [80]. Although the approach to removing all of the listed genera from a dataset has been proven effective [81,82,83], it can still result in the removal of “species” that may be indigenous and ecologically important. In this study, we isolated species that belong to genera identified as contaminants in previous studies [80,84]. These comprise *Brevibacillus*, *Dietzia*, *Kocuria*, *Paenibacillus*, *Paracoccus*, *Pseudomonas*, *Rhodococcus*, and *Sphingobium*. However, a distinction between species should be made to differentiate the false from the true contaminants. Nonetheless, comparing ASVs and 16S rRNA gene sequences from cultured strains can bring evidence. For example, sequences from the strains of *Bacillus cereus* isolated in this study were blasted at 100% sequence similarity with ASVs that were detected in almost all the samples, including the blank extraction controls (Table 3), and were closely related with sequences previously detected in human (LZOD01000018). In this case, we assume that this taxon is likely a contamination. Likewise, the isolated *Serratia* is most likely a contaminant, as it was only detected in the blank extraction controls (Table 3) and was closely related with sequences detected in human (Figure S1). While culture-independent methods may be affected by possible inherent contamination problems of subsurface samples, the question of whether a microorganism is truly indigenous may be answered by a comparison with a culture-dependent approach. Cultured strains may have the advantage of representing microorganisms that are adapted to subsurface conditions.

## 5. Conclusions

To conclude, our study is the first to report the culturable microbial diversity from the basaltic subsurface of Surtsey island. In total, we tested 270 enrichments using different temperatures and media. We isolated 195 strains, representing 42 different genera. Most of the isolates were aerobic and heterotrophic bacteria, and few novel species were isolated. In this perspective, a bacterium isolated in this study, *Rhodothermus bifroesti*, has been characterized [56]. The comparison of our cultured strains and ASVs from 16S rRNA amplicon gene datasets obtained from the same samples [37] revealed that isolates were detected using both culture-dependent (isolation) and -independent methods (metabarcoding). This study provided a valuable supplementary description of the microbial diversity in the subsurface of Surtsey. Both culture-independent and dependent methods are essential to improving our insight into the microbial communities inhabiting such an extreme and complex volcanic environment.

## Figures and Tables

**Table 1:** Sample collection.

Sample ID	Sample Type	Sampling Date	Collection Depth (m b.s.)	Collection Temperature (°C)
16.2	Borehole fluid	9 June 2016	166	54
16.7	Borehole fluid	9 June 2016	160	60
16.8	Borehole fluid	9 June 2016	mix	n.a.
17.1	Borehole fluid	3 August 2017	58	85
17.2	Borehole fluid	3 August 2017	120	116
17.3	Borehole fluid	3 August 2017	150	76
17.4	Borehole fluid	3 August 2017	160	52
17.5	Fumarole	5 August 2017	0	64.2–82.3
17.6	Fumarole	4 August 2017	0	40.8
17.8, 17.9, 17.F	Fumarole	4 August 2017	0	56.1–74.6
17.11	Borehole fluid	6 September 2017	140	116
17.13	Borehole fluid	6 September 2017	280	58
17.14	Borehole fluid	6 September 2017	mix	n.a.
17.15	Borehole fluid	6 September 2017	75	98
17.16	Borehole fluid	5 September 2017	60	90
17.17	Borehole fluid	5 September 2017	80	116
17.18	Borehole fluid	5 September 2017	90	122
17.19	Borehole fluid	5 September 2017	100	124
17.22	Borehole fluid	5 September 2017	160	61
17.23	Borehole fluid	5 September 2017	mix	n.a.
18.1	Borehole fluid	19 September 2018	mix	n.a.
18.2	Borehole fluid	19 September 2018	mix	n.a.
18.3	Borehole fluid	19 September 2018	mix	n.a.
B3	Drill core	10 August 2017	15	15.3
B9	Drill core	11 August 2017	32	30
B24	Drill core	12 August 2017	70	109
B30	Drill core	13 August 2017	87	121
B36	Drill core	14 August 2017	105	123
C55	Drill core	25 August 2017	156	64
C59	Drill core	25 August 2017	167	55
C62	Drill core	25 August 2017	176	44.5
C65	Drill core	25 August 2017	181	37

n.a.: not availble.

**Table 2:** Phylogenetic affiliations of the isolates based on a comparative analysis of their 16S rRNA gene sequences with the SILVA database.

Phylogenetic Phylum or Class	Family or Genus	Sample Origin	Culture Conditions	Number of Strains Isolated	Borehole Fluid	Fumarole	Drill Core
**Actinobacteriota**	*Arthrobacter*	17.9, 18.1	166, O_2_, 22 °C	3	1	2	0
	*Cryobacterium*	16.8	M, O_2_, 40 °C	1	1	0	0
	*Frigoribacterium*	16.8	M, O_2_, 40 °C	1	1	0	0
	*Microbacterium*	B3, C59	166, MB, SO, O_2_, 22 °C	4	0	0	4
	*Dietzia*	B9	MB, O_2_, 22 °C	1	0	0	1
	*Georgenia*	B24	MB, O_2_, 22 °C	1	0	0	1
	*Glutamicibacter*	17.16	166 and MB, O_2_, 22 °C	4	4	0	0
	*Janibacter*	17.3, 16.7	166, O_2_, 22 °C	4	4	0	0
	*Leifsonia*	16.8	M, O_2_, 40 °C	1	1	0	0
	Intrasporangiaceae	17.4	166, O_2_, 22 °C	2	2	0	0
	*Kocuria*	17.15, 17.16	166, O_2_, 22 °C	2	2	0	0
	*Micrococcus*	16.8, B3	M and SO, O_2_, 22 and 40 °C	2	1	0	1
	*Paeniglutamicibacter*	17.17, 17.19, 18.2, 18.3	166 and MB, O_2_, 22 °C	7	7	0	0
	*Rhodococcus* group 1	17.17	MB, O_2_, 22 °C	1	1	0	0
	*Rhodococcus* group 2	17.2, 17.17, 17.22, 18.1	166 and MB, O_2_, 22 °C	5	5	0	0
	*Rubrobacter*	17.15, 17.2, 17.22, B24	166, O_2_, 60 °C	4	3	0	1
				**43**	**33**	**2**	**8**
**Bacteroidota**	*Rhodothermus*	17.15, 17.2, 17.22, B24	166, O_2_, 60 °C	8	6	0	2
				**8**	**6**	**0**	**2**
**Deinococcota**	*Thermus*	17.5, 17.8, 17.9	166, O_2_, 80 °C	4	0	4	0
				**4**	**0**	**4**	**0**
**Firmicutes**	*Bacillus* group 1	17.11, 17.14, 17.15, B9	MB, O_2_, 22 °C	5	5	0	0
	*Bacillus* group 2	17.1	166, O_2_, 22 °C	1	0	0	1
	*Bacillus (para)licheniformis*	17.2, 17.5, 17.8, 17.9, 17.F	166, with and without O_2_, 22, 50 and 60 °C	15	2	13	0
	*Bacillus cereus* group	17.8, 17.15, 17.F, C55, C65	166, with and without O_2_, 22 and 37 °C	5	1	2	2
	*Brevibacillus*	17.8	166, O_2_, 60 °C	2	0	2	0
	*Brevibacillus thermoruber*	17.1, 17.5, 17.9	166, O_2_, 60 °C	4	1	3	0
	*Caldalkalibacillus*	17.1, 17.4	166, O_2_, 60 °C	6	6	0	0
	*Geobacillus*	17.16	166, O_2_, 22 and 60 °C	4	4	0	0
	*Geobacillus thermoleovorans* group	17.5, 17.8, 17.F	166, O_2_, 60 and 65 °C	20	2	18	0
	*Paenibacillus*	B3, C55, C65	166, O_2_, 22 °C	8	0	0	8
	*Planifilum*	18.3	166, O_2_, 60 °C	1	1	0	0
	Planococcaceae	18.3	MB, O_2_, 22 °C	1	1	0	0
	*Planomicrobium*	18.3	166, O_2_, 22 °C	2	2	0	0
	*Ureibacillus*	17.4, 17.6	166, O_2_, 60 °C	4	1	3	0
				**78**	**26**	**41**	**11**
**Alpha-proteobacteria**	*Brevundimonas*	17.16	166, O_2_, 22 °C	1	1	0	0
	*Paracoccus*	C65	MB, O_2_, 22 °C	1	0	0	1
	*Allorhizobium-Neorhizobium-Pararhizobium-Rhizobium*	B9	MB, O_2_, 22 °C	2	0	0	2
	Sphingomonadaceae	B3	YPS, O_2_, 22 °C	1	0	0	1
	Rhodobacteraceae	B3	SO, O_2_, 22 °C	1	0	0	1
				**6**	**1**	**0**	**5**
**Gamma-proteobacteria**	*Acinetobacter*	16.8	M, O_2_, 40 °C	1	1	0	0
	*Halomonas*	17.15, 17.23, 18.2, 18.3	166 and MB, O_2_, 22 °C	11	11	0	0
	*Marinomonas*	17.13, 17.15	166 and MB, O_2_, 22 °C	2	2	0	0
	*Enterobacter*	16.2, 16.7, 17.5, B3, C55, C65	166, M, I and SO, without O_2_, 22 °C	18	5	1	12
	Enterobacteriaceae	17.5	166, O_2_, 22 °C	1	0	1	0
	*Pseudoalteromonas*	17.23	166 and MB, O_2_, 22 °C	7	7	0	0
	*Pseudomonas* group 1	B30, B36, B9, C62	166 and MB, O_2_, 22 °C	8	0	0	8
	*Pseudomonas* group 2	18.2, 18.3	166, O_2_, 22 °C	3	3	0	0
	*Pseudomonas* group 3	17.8	166, O_2_, 22 °C	1	0	1	0
	*Serratia*	17.5	166, O2, 22 °C	1	0	1	0
	*Shewanella*	B9	MB, O_2_, 22 °C	2	0	0	2
	*Polaromonas*	16.8	M, O_2_, 40 °C	1	1	0	0
				**56**	**30**	**4**	**22**
**Total number of isolated strains**				**195**	**96**	**51**	**48**

**Table 3:** Heatmap comparing partial 16S rRNA gene sequences from the isolated strains to ASVs from 16S rRNA amplicon gene sequencing datasets from [37] (EMBL-EBI, accession number PRJEB42339). F, Fumaroles; BF, Borehole fluids; DC, Drill cores; Control, extraction blanks. Values in bold correspond to sequence similarity percentages above 98.65%, potentially indicating that the same isolated species has been detected in the subsurface using culture-independent method.

Phylogenetic Class	Genus	F	BF	DC	Control	Closest Sequence Similarity Percentage (Megablast)	Isolated From
**Actinobacteriota**	*Arthrobacter*	0	1	0	0	97.794	BF and F
	*Dietzia*	0	2	2	0	**99.265–100**	DC
	*Georgenia*	0	0	0	0		DC
	*Glutamicibacter*	0	0	0	1	**100**	BF
	Intrasporangiaceae	3	7	6	1	**100**	BF
	*Kocuria*	0	1	1	0	**99.259**	BF
	*Leifsonia*	0	2	2	0	**98.684**	BF
	*Microbacterium lacus*	0	0	2	1	**99.029**	DC
	*Micrococcus*	1	2	4	2	**99.457–100**	BF and DC
	*Paeniglutamicibacter*	1	1	2	0	**100**	BF
	*Rhodococcus* group 1	1	1	2	1	**100**	BF
	*Rhodococcus* group 2	1	2	2	0	**100**	BF
	*Rubrobacter*	2	0	1	0	93.605	BF and DC
**Bacteroidota**	*Rhodothermus*	0	2	0	0	95.588	BF and DC
**Deinococcota**	*Thermus*	0	4	5	1	**98.693–100**	F
**Firmicutes**	*Bacillus (para)licheniformis*	0	1	3	0	**99.495–100**	BF and F
	*Bacillus cereus*	2	2	2	1	**100**	BF, F and DC
	*Bacillus* group 1	0	0	1	0	97.024	BF and DC
	*Bacillus* group 2	0	0	0	0	/	BF
	*Brevibacillus*	0	1	8	0	**99.052–100**	BF and F
	*Caldalkalibacillus*	0	2	0	1	98.529	BF
	*Geobacillus*	0	2	2	0	**99.074–99.537**	BF
	*Geobacillus thermoleovorans* group	0	2	5	0	**99.487–100**	F
	*Paenibacillus*	0	0	2	0	**100**	DC
	*Planifilum*	0	0	0	0	/	BF
	*Planococcaceae*	0	2	2	0	97.674	BF
	*Planomicrobium*	0	1	0	0	**98.897**	BF
	*Ureibacillus*	0	0	2	0	**100**	F and BF
**Alphaproteobacteria**	*Allorhizobium-Neorhizobium-Pararhizobium-Rhizobium*	0	1	1	0	**99.034**	DC
	*Brevundimonas*	1	1	5	1	**99.457–100**	BF
	*Paracoccus*	0	0	1	0	**100**	DC
	*Rhodobacteraceae*	0	0	3	0	**100**	DC
	*Sphingobium*	2	1	1	0	**99.425–100**	DC
**Gammaproteobacteria**	*Acinetobacter*	4	4	5	2	**99.254–100**	BF
	*Enterobacteriaceae*	2	3	4	2	**99.533–100**	F, BF and DC
	*Halomonas*	0	4	0	0	**99.265–100**	BF
	*Marinomonas*	0	1	3	0	**99.052–100**	BF
	*Polaromonas*	0	2	3	1	98.276	BF
	*Pseudoalteromonas*	2	4	4	1	**100**	BF
	*Pseudomonas* group 1	2	7	3	0	**99.306–100**	DC
	*Pseudomonas* group 2	0	2	2	2	**98.529–98.897**	F
	*Pseudomonas* group 3	3	4	7	0	**98.907–100**	BF
	*Serratia*	0	0	0	1	97.619	F
	*Shewanella*	0	0	1	0	96.691	DC

## Data Availability

Partial 16S rRNA gene sequences obtained in this study have been deposited in the GenBank databases under accession numbers OK534092 to OK534145.

## Supplementary Materials

The following supporting information can be downloaded at https://www.mdpi.com/article/10.3390/microorganisms10061177/s1, Figure S1: Maximum likelihood 16S rRNA gene sequence phylogenetic tree of the cultured strains using ARB; Figure S2: Sampling and workflow of experiment. Table S1: Medium 166 modified from [39] (without proline). Grunnur base from medium 162 from [85]. Table S2: Medium YPS. Table S3: Medium sulfate reducer (SO). Table S4: Medium iron reducer (I). Table S5: Medium methanogen (M). Table S6: Culture collection represented by 55 selected strains.
