# Supplementary material for: Culturable Bacterial Diversity from the Basaltic Subsurface of the Young Volcanic Island of Surtsey, Iceland
Source: Microorganisms. 2022 Jun 8;10(6):1177. doi: 10.3390/microorganisms10061177 (PMC9229223; doi:10.3390/microorganisms10061177)
Supplement: Supplementary file 1 [file microorganisms-10-01177-s001.zip › microorganisms-1751553-supplementary.pdf]

Supplementary

Culturable Bacterial Diversity from the Basaltic Subsurface of the Young Volcanic Island of Surtsey, Iceland

Pauline Bergsten [1,2], Pauline Vannier [1], Julie Frion [1], Alan Mougeolle [1] and Viggó Þór Marteinsson [1,3,4*]

1 Matís, Exploration and Utilization of Genetic Resources, Reykjavík, Iceland
2 Faculty of Life and Environmental Sciences, University of Iceland, Reykjavík, Iceland
3 Faculty of Food Science and Nutrition, University of Iceland, Reykjavík, Iceland
4 Agricultural University of Iceland, Reykjavik, Iceland
* Correspondence: viggo@matis.is

Supplementary text:

Using another method, a supplementary phylogenetic tree was constructed for comparison. The ARB software package (http://www.arb-home.de) (Ludwig et al., 2004) was used with the SILVA SSU Release 138.1 database (Pruesse et al., 2012; Quast et al., 2013) (Fig. S1). Already aligned sequences using SINA were imported in ARB (Fasta_wgap.ift). The sequence alignments were not manually refined. The 151 sequences were added to the SILVA database tree using "ARB parsimony (quick add marked) and the ecoli filter. The closest neighbors were selected (442 sequences) and the tree was built using the sequence data using the maximum likelihood (PhyML-20130708 DNA algorithm) with default parameters. No outgroup was selected.

Supplementary figures:

Figure S1. Maximum likelihood 16S rRNA gene sequence phylogenetic tree of the cultured strains.

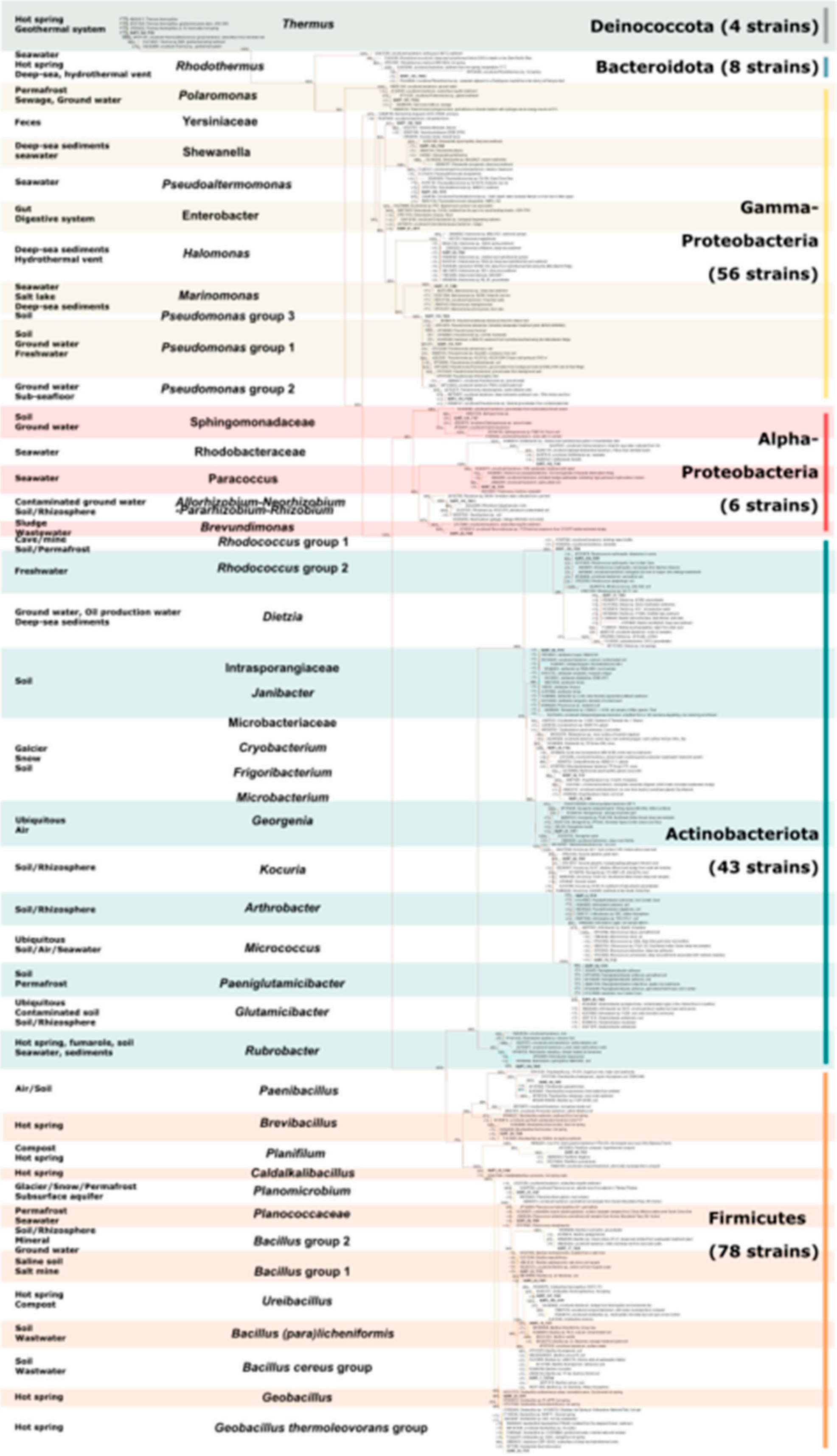

Figure S2. Sampling and workflow of experiment.

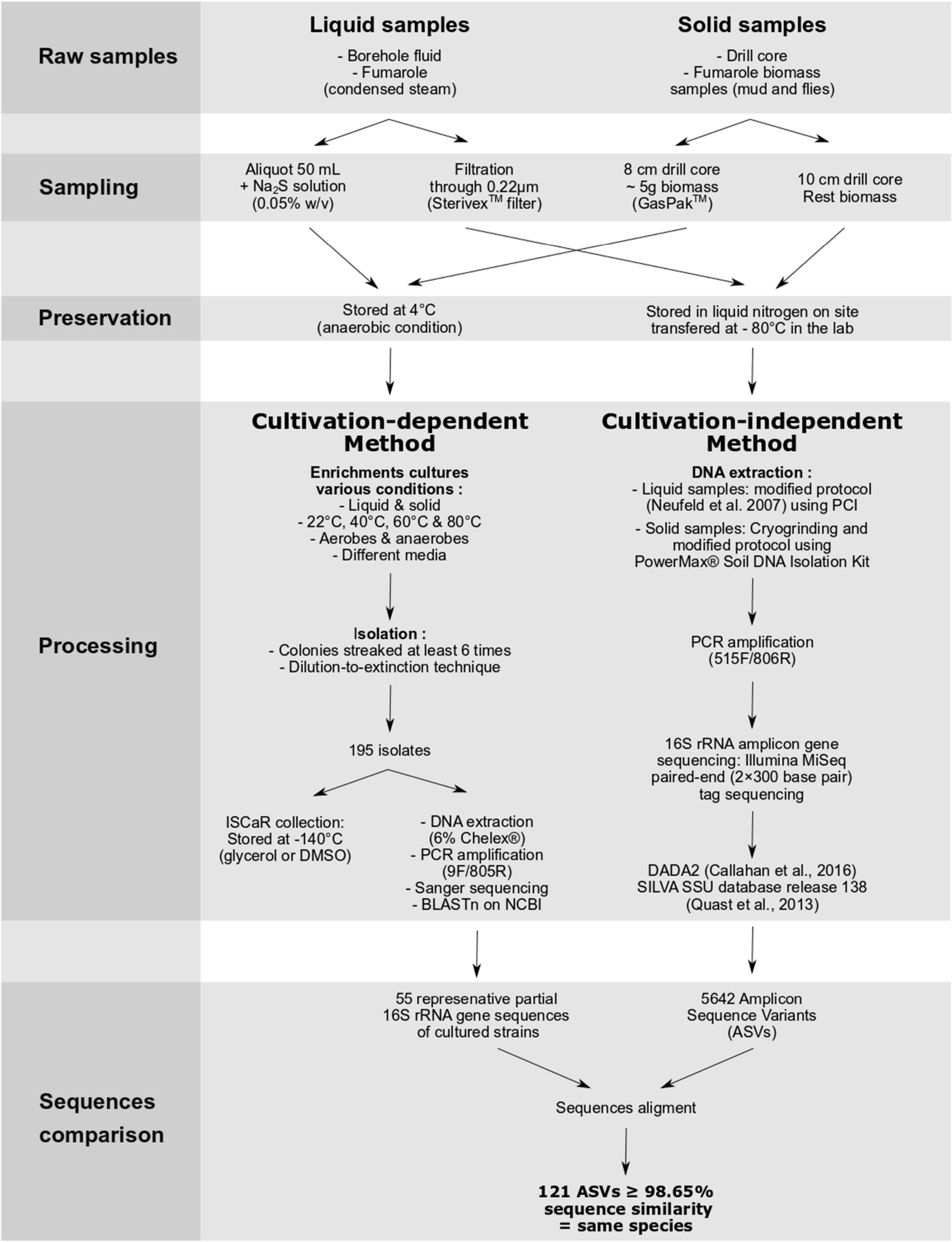

Supplementary tables:

Table S1. Medium 166 modified from (Hjorleifsdottir et al., 2001) (without proline). Grunnur base from medium 162 from (Degryse et al., 1978).

**For 1L of liquid media:**	
NaCl	20 g
K_2HPO_4	0.3 g
Yeast extract	1 g
Peptone	1 g
Tryptone	1 g
Glucose	0.5 g
Amidon (starch)	0.5 g
Na-pyruvate	0.6 g
Na_2CO_3	0.18 g
Base "Grunnur"	100 mL
Hot tap water	900 mL
pH adjusted to 7-7.5	
**Grunnur (for 1L):**	
Titriplex I (nitrilotriacetic acid)	1.32 g
$CaSO_4 \times 2H_2O$	0.4 g
$MgCl_2 \times 6H_2O$	2.0 g
Trace elements (Wolfe´s mineral solution)	5 ml
Ironcitrate solution	5 ml
pH adjusted to 7,2	
**Ironcitrate (for 1L):**	
Na_3citrate x $2H_2O$	2.94 g
$FeCl_3 \times 6H_2O$	2.7 g

Table S2. Medium YPS.

• **Solution A (autoclave)**	For 970 mL
NaCl	10 to 30 g
NH_4Cl	0.5 g
$MgSO_4$ x 7 H_2O	3.4 g
$MgCl_2$ x 6 H_2O	4.18 g
KCl	0.33 g
$FeSO_4$ x 7 H_2O	0.01 g
Na_2SeO_3 x 5 H_2O	1 mg
PIPES	3 g
resazurin (0.1% w/v)	few drops
ASW supplemented by:	
Yeast extract	0.5 g
Peptone	0.5 g
pH at 7	
• **Solution B (autoclave)**	5 mL
K_2HPO_4	0.14 g
• **Solution C (autoclave)**	5 mL
$CaCl_2$ x 2 H_2O	0.50 g
• **Trace element solution (medium 141) (filtration)**	10 mL
• **Extra solutions (filtration)**	
Vitamin solution (see medium 141)	10 mL
elemental sulfur	10 g

After sterilization by autoclave or filtration (0.22 µm) and cooling, the solutions were pooled.

Table S3. Medium Sulfate reducer (SO).

• **Solution A (autoclave)**	For 960 mL
NaCl	10 to 30 g
NH_4Cl	0.5 g
$MgSO_4$ x 7 H_2O	3.4 g
$MgCl_2$ x 6 H_2O	4.18 g
KCl	0.33 g
$FeSO_4$ x 7 H_2O	0.01 g
Na_2SeO_3 x 5 H_2O	1 mg
PIPES	3 g
resazurin (0.1% w/v)	few drops
ASW supplemented by:	
Na_2SO_4	2 g
Yeast extract	0.2 g
L-lactate	0.5 g
Na-pyruvate	0.5 g
L-ascorbate	0.5 g
pH at 7.5	
• **Solution B (autoclave)**	5 mL
K_2HPO_4	0.14 g
• **Solution C (autoclave)**	5 mL
$CaCl_2$ x 2 H_2O	0.50 g
• **Trace element solution (medium 141) (filtration)**	10 mL
• **Extra solutions (filtration)**	
$NaHCO_3$ (0.1% w/v)	20 mL

After sterilization by autoclave or filtration (0.22 µm) and cooling, the solutions were pooled.

Table S4. Medium Iron reducer (I).

• **Solution A (autoclave)**	For 950 mL
NaCl	10 to 30 g
NH_4Cl	0.5 g
$MgSO_4$ x 7 H_2O	3.4 g
$MgCl_2$ x 6 H_2O	4.18 g
KCl	0.33 g
$FeSO_4$ x 7 H_2O	0.01 g
Na_2SeO_3 x 5 H_2O	1 mg
PIPES	3 g
resazurin (0.1% w/v)	few drops
ASW supplemented by:	
Fe(III) citrate	10 g*
Na-acetate	2.5 g
pH at 8	
• **Solution B (autoclave)**	5 mL
K_2HPO_4	0.14 g
• **Solution C (autoclave)**	5 mL
$CaCl_2$ x 2 H_2O	0.50 g
• **Trace element solution (medium 141) (filtration)**	10 mL
• **Extra solutions (filtration)**	
$NaHCO_3$ (0.2% w/v)	20 mL
Vitamin solution (see medium 141)	10 mL
Na_2WO_4 x 2 H_2O (0.1% w/v) (stored under N_2)	0.25 mL

* First dissolve ferric citrate by heating the water under continuous stirring. After cooling to room temperature adjust the pH to 6.0, then add and dissolve the remaining ingredients to prepare solution A. After sterilization by autoclave or filtration (0.22 µm) and cooling, the solutions were pooled.

Table S5. Medium Methanogen (M).

• **Solution A (autoclave)**	For 945 mL
NaCl	10 to 30 g
NH_4Cl	0.5 g
$MgSO_4$ x 7 H_2O	3.4 g
$MgCl_2$ x 6 H_2O	4.18 g
KCl	0.33 g
$FeSO_4$ x 7 H_2O	0.01 g
Na_2SeO_3 x 5 H_2O	1 mg
PIPES	3 g
resazurin (0.1% w/v)	few drops
ASW supplemented by:	
Yeast extract	0.2 g
pH at 7.5	
• **Solution B (autoclave)**	5 mL
K_2HPO_4	0.14 g
• **Solution C (autoclave)**	5 mL
$CaCl_2$ x 2 H_2O	0.50 g
• **Trace element solution (medium 141) (filtration)**	10 mL
• **Extra solutions (filtration)**	
$NaHCO_3$ (0.2% w/v)	20 mL
Vitamin solution (see medium 141)	10 mL
Methanol	5 mL
Coenzyme M (stored under N_2)	0.5 g/L

After sterilization by autoclave or filtration (0.22 µm) and cooling, the solutions were pooled.

Table S6. 16S rRNA gene sequences from the 55 selected cultured strains representing the culture collection.

Seq Num	ISCAR num	Accession number (Genbank)	Table 2 groups (SILVA)	bps	Blast seq	Description NCBI	Query cover	Per. Identity (BLASTn)	lca_tax_slv
1	7133	did not passed quality	Acinetobacter	147	>NR_113346.1	Acinetobacter lwoffii strain JCM 6840	0,99	1	Bacteria;Proteobacteria;Gammaproteobacteria;Pseudomonadales;Moraxellaceae;Acinetobacter;
4	7313	OK534092	Arthrobacter	810	>NR_041546.1	Arthrobacter humicola strain KV-653	1	0,9975	Bacteria;Actinobacteriota;Actinobacteria;Micrococcales;Micrococcaceae;Arthrobacter;
7	7357	OK534093	Bacillus cereus	534	>NR_121761.1	Bacillus toyonensis strain BCT-7112	1	1	Bacteria;Firmicutes;Bacilli;Bacillales;Bacillaceae;Bacillus;
13	7211	OK534094	Bacillus (para)licheniformis	453	>NR_118996.1	Bacillus licheniformis strain DSM 13	1	1	Bacteria;Firmicutes;Bacilli;Bacillales;Bacillaceae;Bacillus;
17	7236	OK534095	Bacillus group 2	446	>NR_025741.1	Bacillus patagoniensis strain PAT 05	1	1	Bacteria;Firmicutes;Bacilli;Bacillales;Bacillaceae;Bacillus;
22	7378	OK534096	Bacillus group 1	448	>NR_026139.1	Bacillus pseudofirmus strain DSM 8715	1	0,9912	Bacteria;Firmicutes;Bacilli;Bacillales;Bacillaceae;Bacillus;
27	7207	OK534097	Brevibacillus thermoruber	444	>NR_112213.1	Brevibacillus thermoruber strain DSM 7064	1	0,9977	Bacteria;Firmicutes;Bacilli;Brevibacillales;Brevibacillaceae;Brevibacillus;
28	7309	OK534098	Brevibacillus	552	>NR_112213.1	Brevibacillus thermoruber strain DSM 7064	0,99	0,9982	Bacteria;Firmicutes;Bacilli;Brevibacillales;Brevibacillaceae;Brevibacillus;
29	7368	OK534099	Brevundimonas	536	>NR_113586.1	Brevundimonas vesicularis strain NBRC 12165	1	0,9981	Bacteria;Proteobacteria;Alphaproteobacteria;Caulobacterales;Caulobacteraceae;Brevundimonas;

35	7238	OK534100	Caldalkalibacillus	543	>NR_043653.1	Caldalkalibacillus uzonensis strain JW/WZ-YB58	1	0,9945	Bacteria;Firmicutes;Bacilli;Caldalkalibacillales;Caldalkalibacillaceae;Caldalkalibacillus;
36	7134	OK534101	Cryobacterium	528	>NR_170455.1	Cryobacterium soli strain GCJ02	1	0,9924	Bacteria;Actinobacteriota;Actinobacteria;Micrococcales;Microbacteriaceae;Cryobacterium;
37	7393	OK534102	Dietzia	1160	>NR_117963.1	Dietzia cercidiphylli strain X0053	1	1	Bacteria;Actinobacteriota;Actinobacteria;Corynebacteriales;Dietziaceae;Dietzia;
38	7131	OK534103	Frigoribacterium	473	>NR_115033.1	Frigoribacterium faeni strain DSM 10309	1	0,9979	Bacteria;Actinobacteriota;Actinobacteria;Micrococcales;Microbacteriaceae;Frigoribacterium;
39	7191	OK534104	Geobacillus thermoleovorans group	353	>NR_114089.1	Geobacillus kaustophilus NBRC 102445	1	1	Bacteria;Firmicutes;Bacilli;Bacillales;Bacillaceae;Geobacillus;
41	7371	OK534105	Geobacillus	533	>NR_132400.1	Geobacillus subterraneus subsp. aromaticivorans strain Ge1	0,99	0,9962	Bacteria;Firmicutes;Bacilli;Bacillales;Bacillaceae;Geobacillus;
51	7472	OK534106	Georgenia	484	>NR_112820.1	Georgenia muralis strain NBRC 103560	1	0,9877	Bacteria;Actinobacteriota;Actinobacteria;Micrococcales;Bogoriellaceae;Georgenia;
55	7360	OK534107	Glutamicibacter	553	>NR_025612.1	Glutamicibacter bergerei strain Ca106	1	1	Bacteria;Actinobacteriota;Actinobacteria;Micrococcales;Micrococcaceae;Glutamicibacter;
56	7319	OK534108	Halomonas	1231	>NR_114866.1	Halomonas glaciei strain DD 39	1	0,9903	Bacteria;Proteobacteria;Gammaproteobacteria;Pseudomonadales;Halomonadaceae;Halomonas;
61	7458	OK534109	Halomonas	544	>NR_027185.1	Halomonas sulfidaeris Esulfide1	1	0,9818	Bacteria;Proteobacteria;Gammaproteobacteria;Pseudomonadales;Halomonadaceae;Halomonas;
62	7320	OK534110	Halomonas	513	>NR_027185.1	Halomonas sulfidaeris Esulfide1	1	0,9787	Bacteria;Proteobacteria;Gammaproteobacteria;Pseudomonadales;Halomonadaceae;Halomonas;

66	7314	OK534111	Janibacter	522	>NR_026362.1	Janibacter limosus strain DSM 11140	1	0,9981	Bacteria;Actinobacteriota;Actinobacteria;Micrococcales;Intrasporangiaceae;Janibacter;
68	7364	OK534112	Kocuria	566	>NR_026451.1	Kocuria palustris strain TAGA27	1	0,9929	Bacteria;Actinobacteriota;Actinobacteria;Micrococcales;Micrococcaceae;Kocuria;
69	7190	OK534113	Enterobacteriaceae	230	> NR_118121.1	Pantoea rwandensis strain LMG 26275	1	1	Bacteria;Proteobacteria;Gammaproteobacteria;Enterobacterales;Enterobacteriaceae;
70	7130	OK534114	Leifsonia	341	>NR_115031.1	Leifsonia poae strain DSM 15202	1	1	Bacteria;Actinobacteriota;Actinobacteria;Micrococcales;Microbacteriaceae;Leifsonia;
71	7386	OK534115	Marinomonas	526	>NR_116234.1	Marinomonas foliarum strain IVIA-Po-155	1	0,9924	Bacteria;Proteobacteria;Gammaproteobacteria;Pseudomonadales;Marinomonadaceae;Marinomonas;
73	7392	OK534116	Microbacterium lacus	531	>NR_041516.1	Microbacterium ginsengisoli strain Gsoil 259	0,99	0,9811	Bacteria;Actinobacteriota;Actinobacteria;Micrococcales;Microbacteriaceae;Microbacterium;
76	7395	OK534117	Microbacterium lacus	1356	>NR_041563.1	Microbacterium lacus strain A5E-52	1	0,9897	Bacteria;Actinobacteriota;Actinobacteria;Micrococcales;Microbacteriaceae;Microbacterium;
78	7132	OK534118	Micrococcus	342	>NR_117194.1	Micrococcus cohnii strain WS4601	1	0,9942	Bacteria;Actinobacteriota;Actinobacteria;Micrococcales;Micrococcaceae;Micrococcus;
80	7209	OK534119	Intrasporangiaceae	196	> NR_164959.1	Janibacter massiliensis strain Marseille-P4121	1	1	Bacteria;Actinobacteriota;Actinobacteria;Micrococcales;Intrasporangiaceae;
82	7251	OK534120	Paenibacillus	534	>NR_113987.1	Paenibacillus pasadenensis strain NBRC 101214	1	0,9981	Bacteria;Firmicutes;Bacilli;Paenibacillales;Paenibacillaceae;Paenibacillus;

90	7379	OK534121	Paeniglutamicibacter	559	>NR_026237.1	Paeniglutamicibacter sulfureus strain DSM 20167	1	0,9946	Bacteria;Actinobacteriota;Actinobacteria;Micrococcales;Micrococcaceae;Paeniglutamicibacter;
91	7271	OK534122	Enterobacter	598	>NR_111998.1	Pantoea agglomerans strain JCM1236	1	1	Bacteria;Proteobacteria;Gammaproteobacteria;Enterobacterales;Enterobacteriaceae;Enterobacter;
92	7310	OK534123	Paracoccus	560	>NR_113921.1	Paracoccus marinus strain NBRC 100637	1	0,9964	Bacteria;Proteobacteria;Alphaproteobacteria;Rhodobacterales;Rhodobacteraceae;Paracoccus;
94	7312	OK534124	Janibacter	471	>NR_108472.1	Phycicoccus badiiscoriae strain Sco-B23	1	1	Bacteria;Actinobacteriota;Actinobacteria;Micrococcales;Intrasporangiaceae;Janibacter;
95	7331	OK534125	Planifilum	1375	>NR_043563.1	Planifilum yunnanense strain LA5	1	0,9993	Bacteria;Firmicutes;Bacilli;Thermoactinomycetales;Thermoactinomycetaceae;Planifilum;
96	7456	OK534126	Planococcaceae	407	>NR_156838.1	Planococcus versutus strain L10.15	1	0,978	Bacteria;Firmicutes;Bacilli;Bacillales;Planococcaceae;
97	7327	OK534127	Planomicrobium	1189	>NR_113593.1	Planomicrobium okeanokoites strain NBRC 12536	1	0,9975	Bacteria;Firmicutes;Bacilli;Bacillales;Planococcaceae;Planomicrobium;
103	7375	OK534128	Pseudoalteromonas	592	>NR_114191.1	Pseudoalteromonas undina strain NBRC 103039	1	1	Bacteria;Proteobacteria;Gammaproteobacteria;Enterobacterales;Pseudoalteromonadaceae;Pseudoalteromonas;
104	7391	OK534129	Pseudoalteromonas	549	>NR_114191.2	Pseudoalteromonas undina strain NBRC 103040	1	1	Bacteria;Proteobacteria;Gammaproteobacteria;Enterobacterales;Pseudoalteromonadaceae;Pseudoalteromonas;

110	7352	OK534130	Pseudomonas group 1	603	>NR_025103.1	Pseudomonas brenneri strain CFML 97-391	1	1	Bacteria;Proteobacteria;Gammaproteobacteria;Pseudomonadales;Pseudomonadaceae;Pseudomonas;
114	7205	OK534131	Pseudomonas group 3	365	>NR_152710.1	Pseudomonas turukhanskensis strain IB1.1	1	0,9945	Bacteria;Proteobacteria;Gammaproteobacteria;Pseudomonadales;Pseudomonadaceae;Pseudomonas;
116	7329	OK534132	Pseudomonas group 2	1218	>NR_134795.1	Pseudomonas zhaodongensis strain NEAU-ST5-21	1	0,9984	Bacteria;Proteobacteria;Gammaproteobacteria;Pseudomonadales;Pseudomonadaceae;Pseudomonas;
119	7381	OK534133	Allorhizobium-Neorhizobium-Pararhizobium-Rhizobium	557	>NR_116445.1	Rhizobium rosettiformans W3	1	0,9911	Bacteria;Proteobacteria;Alphaproteobacteria;Rhizobiales;Rhizobiaceae;Allorhizobium-Neorhizobium-Pararhizobium-Rhizobium;
120	7396	OK534134	Rhodococcus group 1	512	>NR_116275.1	Rhodococcus cercidiphylli strain YIM 65003	1	1	Bacteria;Actinobacteriota;Actinobacteria;Corynebacteriales;Nocardiaceae;Rhodococcus;
124	7354	OK534135	Rhodococcus group 2	494	>NR_145886.1	Rhodococcus degradans strain CCM 4446	1	1	Bacteria;Actinobacteriota;Actinobacteria;Corynebacteriales;Nocardiaceae;Rhodococcus;
129	7401	OK534136	Rhodothermus	1255	>NR_074728.1	Rhodothermus marinus DSM 4252	1	0,9536	Bacteria;Bacteroidota;Rhodothermia;Rhodothermales;Rhodothermaceae;Rhodothermus;
134	7405	OK534137	Rubrobacter	578	>NR_074552.1	Rubrobacter xylanophilus strain DSM 9941	1	0,9692	Bacteria;Actinobacteriota;Rubrobacteria;Rubrobacterales;Rubrobacteriaceae;Rubrobacter;
138	7206	OK534138	Serratia	420	>NR_025339.1	Serratia fonticola strain DSM 4576	1	0,9976	Bacteria;Proteobacteria;Gammaproteobacteria;Enterobacterales;Yersiniaceae;Serratia;

140	7394	OK534139	Shewanella	1247	>NR_040951.1	Shewanella kaireitica strain c931	1	0,9912	Bacteria;Proteobacteria;Gammaproteobacteria;Enterobacterales;Shewanellaceae;Shewanella;
141	7137	OK534140	Sphingomonadaceae	294	>NR_026304.1	Sphingobium xenophagum strain BN6	1	1	Bacteria;Proteobacteria;Alphaproteobacteria;Sphingomonadales;Sphingomonadaceae;
142	7145	OK534141	Rhodobacteraceae	404	>NR_043547.1	Sulfitobacter litoralis strain Iso 3	1	1	Bacteria;Proteobacteria;Alphaproteobacteria;Rhodobacterales;Rhodobacteraceae;
143	7204	OK534142	Thermus	278	>NR_037066.1	Thermus thermophilus HB8	1	1	Bacteria;Deinococcota;Deinococci;Thermales;Thermaceae;Thermus;
144	7189	OK534143	Thermus	317	>NR_037066.1	Thermus thermophilus HB8	1	1	Bacteria;Deinococcota;Deinococci;Thermales;Thermaceae;Thermus;
150	7215	OK534144	Ureibacillus	480	>NR_119203.1	Ureibacillus thermosphaericus strain P-11	1	1	Bacteria;Firmicutes;Bacilli;Bacillales;Bacillaceae;Ureibacillus;
151	7129	OK534145	Polaromonas	251	>NR_109102.1	Variovorax defluvii strain 2C1-b	1	0,9801	Bacteria;Proteobacteria;Gammaproteobacteria;Burkholderiales;Comamonadaceae;Polaromonas;
